# Orbital Mapping of Semiconducting Perylenes on Cu(111)

**DOI:** 10.1021/acs.jpcc.1c05575

**Published:** 2021-10-28

**Authors:** Giovanni Di Santo, Tanja Miletić, Mathias Schwendt, Yating Zhou, Benson M. Kariuki, Kenneth D. M. Harris, Luca Floreano, Andrea Goldoni, Peter Puschnig, Luca Petaccia, Davide Bonifazi

**Affiliations:** † Elettra Sincrotrone Trieste, Strada Statale 14 km 163.5, 34149 Trieste, Italy; ‡ School of Chemistry, Cardiff University, Park Place, CF10 3AT Cardiff, U.K.; § Institute of Physics, University of Graz, NAWI Graz, 8010 Graz, Austria; ∥ CNR-IOM Laboratory, TASC in Area Science Park, s.s. 14, km 163.5, 34149 Trieste, Italy; ⊥ Institute of Organic Chemistry, University of Vienna, Währinger Str. 38, 1090 Vienna, Austria

## Abstract

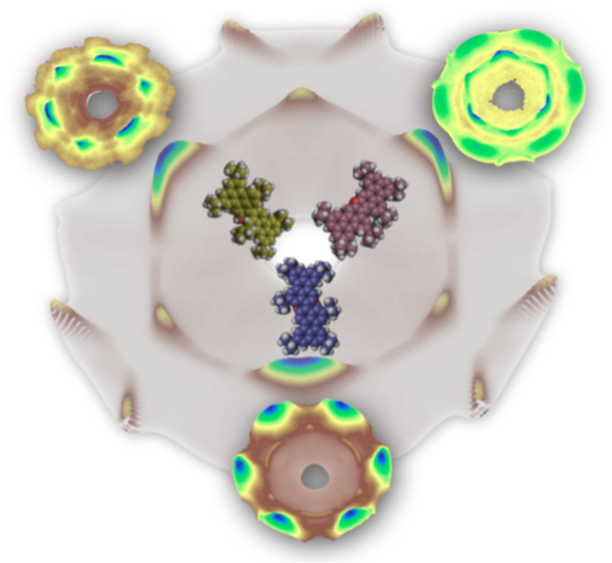

Semiconducting O-doped polycyclic aromatic hydrocarbons constitute a class of molecules whose optoelectronic properties can be tailored by acting on the π-extension of the carbon-based frameworks and on the oxygen linkages. Although much is known about their photophysical and electrochemical properties in solution, their self-assembly interfacial behavior on solid substrates has remained unexplored so far. In this paper, we have focused our attention on the on-surface self-assembly of O-doped bi-perylene derivatives. Their ability to assemble in ordered networks on Cu(111) single-crystalline surfaces allowed a combination of structural, morphological, and spectroscopic studies. In particular, the exploitation of the orbital mapping methodology based on angle-resolved photoemission spectroscopy, with the support of scanning tunneling microscopy and low-energy electron diffraction, allowed the identification of both the electronic structure of the adsorbates and their geometric arrangement. Our multi-technique experimental investigation includes the structure determination from powder X-ray diffraction data for a specific compound and demonstrates that the electronic structure of such large molecular self-assembled networks can be studied using the reconstruction methods of molecular orbitals from photoemission data even in the presence of segregated chiral domains.

## Introduction

**Figure 1:**
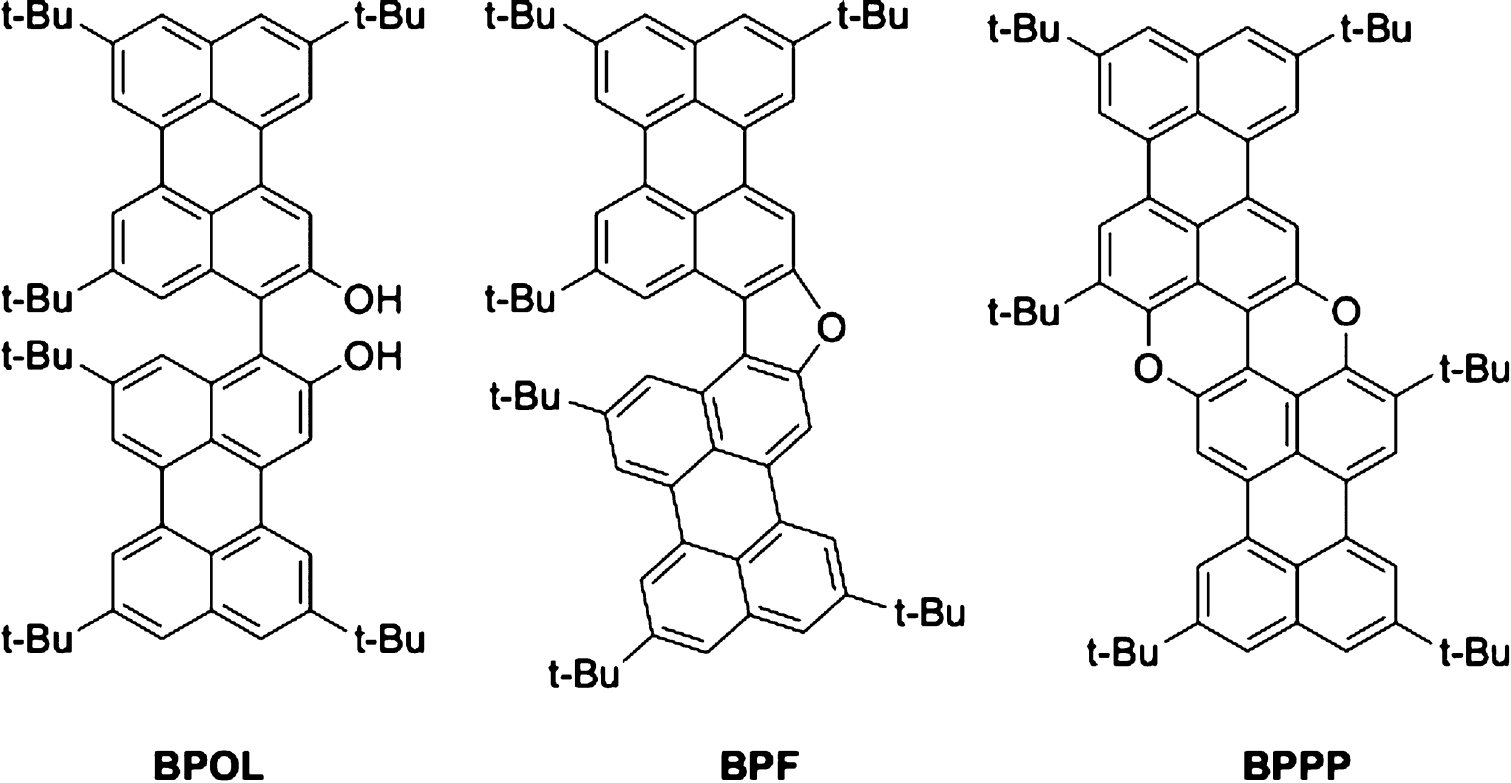
Chemical structures of the O-doped perylenes investigated in this study: 5,5′,8,8′,11,11′-hexa-*tert*-butyl-[3,3′-biperylene]-2,2′-diol (**BPOL**), 2,5,8,14,17,20-hexa-*tert*-butyldiperyleno[2,3-*b*:3′,2′-*d*]furan (**BPF**), and 2,5,9,12,15,19-hexa-*tert*-butylbenzo[5′,10′]anthra[9′,1′,2′:7,8,1]isochromeno[5,4,3-*cde*]benzo[5,10]anthra[9,1,2-*hij*]isochromene (**BPPP**).^23^

Recently reported semiconducting O-doped polycyclic aromatic hydrocarbons (PAHs), bearing a pyranopyranil or a furanyl core, are very appealing candidates for photoelectronic applications and as p-type semiconductors, showing exceptionally high emission yields and tunable optoelectronic properties in solution.^1−5^ Photophysical and electrochemical characterization showed that complementary spectroscopic and redox properties could be tailored through fine tuning of both the π-extension of the carbon scaffold and the oxygen linkages (i.e., furanyl vs pyranopyranyl rings). However, without detailed information about their interfacial electronic properties, the implementation of these complexes into devices would be based on a trial and error method because the coupling to a counter electrode may change the electronic and structural properties of the molecular contact layer, ultimately affecting the efficiency of charge transport of the next molecular layers. On one hand, the morphology of the interface governs the degree of order and orientation of the next organic layers,^6−8^ ultimately affecting the contact resistance.^9^ On the other hand, the molecular orientation and degree of rehybridization of the first contact layer determine the intrinsic efficiency of charge injection,^10^ in terms of alignment of conduction bands and molecular orbitals, as well as chemical and spatial localization of the preferential channel of charge transfer. A combination of structural, morphological, and spectroscopic studies of the interfacial layer at metal substrates is necessary to get insight into the mechanisms governing the formation of thin films: from their assembly to the degree of electronic coupling to the substrate.^11−13^ From this perspective, the use of the orbital mapping based on angle-resolved photoemission (ARPES), together with the support of complementary techniques, such as scanning tunneling microscopy (STM) and low-energy electron diffraction (LEED), allows us to shed light on π-conjugated systems, identifying both the electronic structure of the adsorbates and their geometric arrangement.^14^ The momentum distribution of the photoemission intensity can be used to study the orientation of molecules within their unit cell and to disentangle the valence band states stemming from the substrate from those localized on molecules and hence to evaluate the intermolecular and molecule–substrate interactions.^15^ A number of adsorption effects have been derived from ARPES momentum mapping. For instance, chemical modifications in the molecule have been identified,^16,17^ and in several studies, geometrical parameters of the adsorbate could be determined from the orbital momentum mapping including azimuthal orientations of molecules,^18,19^ inter-ring torsion angles,^20,21^ and molecular tilt angles.^22^ Our approach has been to perform a multi-technique experimental investigation of complex PAH molecules with similar geometries. In particular, within the class of biperylene derivatives, we investigated the monolayer phase on the Cu(111) surface of three compounds very similar in elemental composition, but with a substantial difference in their morphological adaptation. The Esatertbutyl-Biperylenol (**BPOL**), Esatertbutyl-Biperyleno-Furanyl (**BPF**), and Esatertbutyl-Biperyleno-Pyranopyranyl (**BPPP**) molecules are basically the same in terms of stoichiometry, with a different linkage between the two perylene arms (see molecular schemes in Figure 1), with one carbon bridge and two OH groups in **BPOL** and one and two bridging oxygen atoms in **BPF** and **BPPP**, respectively. These differences make them behave very differently in terms of optical emission yield and absorption in the UV–visible spectral region.^23^ A different self-organization and intermolecular arrangement is also expected when deposited onto the substrate surface, and as a consequence, the resulting two-dimensional (2D) networks may display distinct electronic features.

Hereafter, we show that O-doped biperylene derivatives deposited on Cu(111) surfaces self-organize into ordered extended homochiral domains with a well-defined molecular lattice. This makes these systems suitable to be studied with the orbital tomography methodology that maps their electronic states in *k*-space. Capitalizing on the ARPES-based momentum mapping methodology, the electronic states of this class of molecules has been probed, showing that they remain intact upon deposition on the surface.

## Experimental and Computational Details

While the crystal structures of **BPOL** and **BPF** were established previously by single-crystal X-ray diffraction analysis,^23,24^ the growth of single crystals of **BPPP** suitable for single-crystal X-ray diffraction turned out to be difficult. Thus, we have carried out structure determination of **BPPP** directly from powder X-ray diffraction (XRD) data. The microcrystalline powder sample of **BPPP** used in this study was prepared by sublimation. High-quality powder XRD data of **BPPP** suitable for structure determination were recorded on a Bruker D8 instrument (Ge-monochromated Cu Kα_1_ radiation; λ = 1.54056 Å) operating in transmission mode. The powder sample of **BPPP** was packed into two capillaries which were flame-sealed and attached to the disc sample holder of the powder XRD instrument. The powder XRD data were recorded over the 2θ range of 4–70° (step size, 0.017°) with a total data collection time of 64 h 4 min. In conjunction with structure determination from powder XRD data, periodic DFT-D calculations involving geometry optimization (with fixed unit cell) were carried out on trial structures at various stages of the structure determination process using the program CASTEP^25^ (Academic Release version 7.02). The periodic DFT-D calculations were run with a basis set cutoff energy of 700 eV, ultrasoft pseudopotentials, the PBE functional, semiempirical dispersion corrections (TS correction scheme), fixed unit cell, preserved space group symmetry, and periodic boundary conditions. The convergence criteria for geometry optimization were 0.01 eV Å^–1^ for forces, 0.00001 eV per atom for energy, and 0.001 Å for atomic displacements.

**BPOL**, **BPF**, and **BPPP** molecules were sublimed in ultra-high vacuum (UHV) from a homemade multiple slot Ta-crucible evaporator at approximately 570, 590, and 410 K, respectively, with a deposition rate of ∼0.1 ML/min. The Cu(111) surface was cleaned by repeated cycles of Ar^+^ sputtering at 1.0 keV at room temperature and thermal annealing up to 900 K.

STM is a technique capable of directly visualizing spatial patterns of the local density of states (LDOS) from the long range (hundreds of nm) down to the sub-nanometer scale, where intra-molecular features can also be revealed with sub-Å resolution.^26^ STM measurements have been performed in UHV (base pressure 3 × 10^–10^ mbar) at the IOM-Elettra joint laboratory for microscopy OSMOS with a SPECS-Aarhus type microscope. The topographic images were obtained at room temperature with a tungsten tip after deposition of increasing coverage up to the full saturation of the monolayer. The length scale was calibrated on atomically resolved images of highly oriented pyrolytic graphyte (HOPG).

LEED and ARPES measurements were carried out at the BaDElPh beamline^27^ of Elettra Synchrotron in Trieste. Using LEED, we obtain additional information on the ordering of a molecular adsorbate with respect to the substrate as it images the periodic pattern in the 2D reciprocal space of the investigated surface. Since the probe depth of the impinging electrons is on the nanometer scale, a LEED pattern contains spots relating to the periodicities of both the substrate and the adsorbate. Moreover, since the LEED probed area is normally of mm size, all domain orientations are generally present in a LEED image. LEED diffractograms have been obtained in situ using beam energies in the range of 16–30 eV, and the best-contrast criterion has been used to calibrate the monolayer coverage. The superlattice parameters have been obtained through the best-match comparison of the experimental patterns with the simulated patterns^a^ (with the starting input of the vectors measured by STM).

When performing photoemission experiments on prototype organic semiconductors, the initial state can be approximated as if the photoelectron stems from a single molecular orbital.^28−30^ The further assumption that the final state is a plane wave (PW) leads to a direct interpretation of ARPES experiments: the transition matrix element reduces to the Fourier transform ϕ̃_*i*_(**k**) of the molecular orbital ϕ_*i*_(**r**) from which the electron is emitted. Thus, the photoemission intensity, *I*(*k*_*x*_,*k*_*y*_) ∝ |**A**·**k**|^2^|ϕ̃_*i*_(**k**)|_*k*=const_^2^, reveals the structure of orbital densities on a hemisphere of constant kinetic energy in momentum space.^29,31^ The selection of a particular value for  allows the extraction of energy distribution curves (EDCs) from the photoemission data cube, facilitating the identification of molecule-derived states. Using horizontal (p) polarized radiation and a photon energy of 31 eV, we have followed the photoelectrons dispersion of the molecular features in the valence band by scanning the Brillouin zone (BZ). The spectra were recorded at polar angles in the range of 6–48° with respect to the surface normal and by azimuthal scans in the range of 0–180°. The resulting converted images from kinetic energy and angular to binding energy and parallel wave vector coordinates have been symmetrized according to the substrate’s *p*3*m*1 symmetry group. The overall energy and angular resolution were set to 100 meV and less than 0.3°, respectively.

For ab initio calculations of the relaxed gas-phase molecules, we employed the VASP code^32−35^ using a sufficiently large unit cell and Γ-point sampling of the Brillouin zone. Exchange–correlation effects were approximated using the generalized gradient approximation.^36^ With the projector augmented wave approach,^37^ this enabled using a relatively low kinetic-energy cutoff of about 500 eV. The molecular orbitals (MOs) were then calculated with NWchem.^38^ The placement of the molecules (and the number of molecules per unit cell) relative to (the symmetry elements of) the substrate was inferred from the STM and LEED measurements. Also, guided by the STM measurements, we included the chiral forms of **BPOL**, **BPF** (intrinsic chirality) and **BPPP** (pro-chirality) in the simulation.

## Results and Discussion

### XRD on BPPP

**Figure 2:**
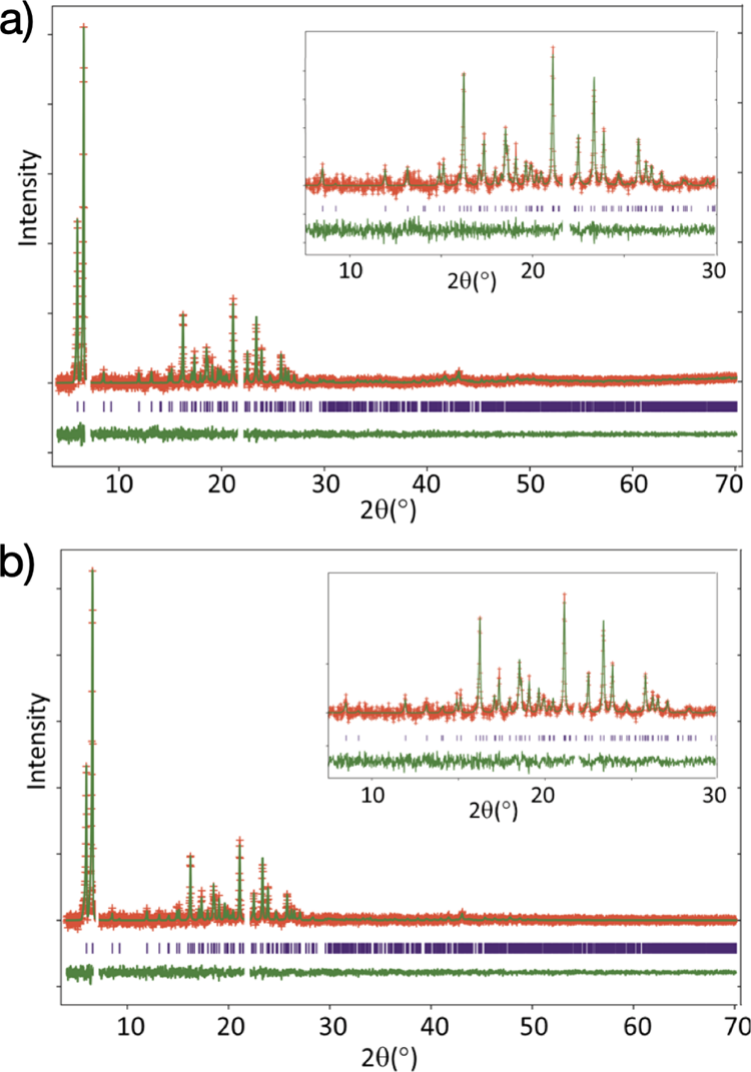
Results from (a) Pawley fitting and (b) final Rietveld refinement of the powder XRD data (background subtracted) for **BPPP** (red “+” marks, experimental data; green line, calculated data; purple tick marks, peak positions; the green line at the bottom shows the difference between the experimental and calculated powder XRD data). In each case, the inset shows an expanded region of the powder XRD data in the range of 2θ = 7.5–30°. Excluded regions correspond to the positions of two impurity peaks, as discussed in the text.

The powder XRD pattern of **BPPP** was indexed using the program DICVOL within the CRYSFIRE suite of indexing programs,^39^ giving the following unit cell with monoclinic metric symmetry: *a* = 13.51 Å, *b* = 5.98 Å, *c* = 29.77 Å, β = 94.7° (*V* = 2395 Å^3^). In the indexing process, two peaks (at 2θ ≈ 4.1° and 2θ ≈ 23.6°) were removed as they were identified as originating from impurity crystalline phases. Given the volume of the unit cell and consideration of density, the number of formula units in the unit cell was assigned as *Z* = 2. From systematic absences, the space group was assigned as *P*2_1_/*c*. As this space group has a multiplicity of 4, the number of molecules of **BPPP** in the asymmetric unit is *Z*′ = 1/2. Profile fitting and unit cell refinement using the Pawley method^40^ in the GSAS-II program^41^ gave a good quality of fit (*R*_wp_ = 2.26%, *R*_p_ = 1.56%; Figure 2a). The refined unit cell and profile parameters obtained from the Pawley fitting procedure were then used in the subsequent structure solution calculations.

Structure solution was carried out using the direct-space genetic algorithm (GA) technique incorporated in the program EAGER,^42−44^ which has been applied to solve the crystal structures of materials from a wide range of areas of application, including materials of biological relevance^45−48^ (e.g., amino acids and peptides), framework structures,^49^ products from solid-state reactions,^50^ optoelectronic materials,^51^ multi-component organic materials,^52,53^ and polymorphic systems.^54^ In the GA structure solution calculation, the centrosymmetric **BPPP** molecule was fixed at a crystallographic inversion center, and thus, the asymmetric unit corresponds to half the molecule, in line with the assignment *Z*′ = 1/2 discussed above. The molecule was constructed using standard bond lengths and bond angles, and trial crystal structures were defined by a total of six structural variables: three orientational variables (corresponding to rotation of the whole molecule around the inversion center) and three torsion-angle variables (corresponding to rotation of each of the three independent *tert*-butyl groups in the asymmetric unit around the C–C(CH_3_)_3_ bond linking the *tert*-butyl group to the aromatic ring). Each GA structure solution calculation involved the evolution of 100 generations for a population of 100 structures, with 10 mating operations and 50 mutation operations carried out per generation. In total, 16 independent GA calculations were carried out, with the same good-quality structure solution obtained in 9 cases.

The best structure solution (i.e., the trial structure with the lowest *R*_wp_ obtained in the GA structure solution calculations) was used as the initial structural model for a geometry optimization calculation using periodic DFT-D methodology (with fixed unit cell), carried out using the CASTEP program.^25^ The crystal structure obtained following the DFT-D geometry optimization was used as the initial structural model for Rietveld refinement, which was carried out using the GSAS-II program.^41^ Standard restraints were applied to bond lengths (74 restraints) and bond angles (151 restraints), and planar restraints were applied to the aromatic ring system (4 restraints). The values of the geometric restraints were derived from the molecular geometry obtained in the DFT-D geometry optimization calculation carried out prior to Rietveld refinement. The final Rietveld refinement gave a good fit to the powder XRD data (*R*_wp_ = 2.29%, *R*_p_ = 1.60%; Figure 2b), comparable to the quality of fit obtained in the profile-fitting procedure using the Pawley method described above, with the following final refined parameters: *a* = 13.4973(8) Å, *b* = 5.9743(5) Å, *c* = 29.7502(28) Å, β = 94.627(10)°; *V* = 2391.13(19) Å^3^ (2θ range, 4–70°; 3867 profile points; 204 refined variables). As final validation, a further periodic DFT-D geometry optimization calculation (with fixed unit cell) was carried out on the crystal structure obtained in the Rietveld refinement. This calculation led to only very minor atomic displacements (rmsd = 0.19 Å for non-hydrogen atoms), confirming that the structure obtained in the final Rietveld refinement is very close to an energy minimum and structurally reasonable.

**Figure 3:**
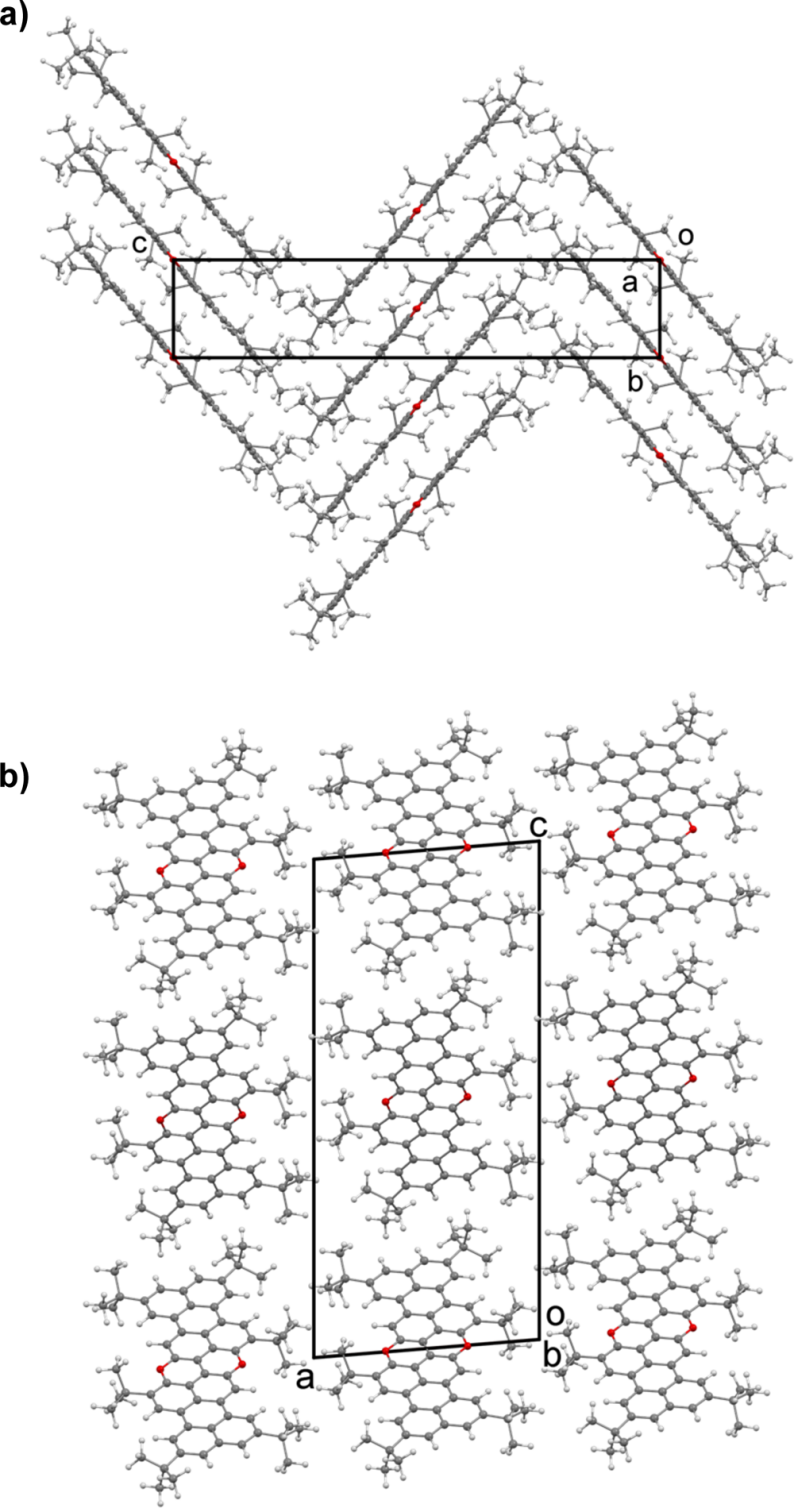
Crystal structure of **BPPP** viewed along (a) *a*-axis and (b) *b*-axis.

The final refined crystal structure of **BPPP** is shown in Figure 3 (the cif file for this structure has been deposited in the Cambridge Structural Database; deposition number, 2078994). In the crystal structure, the molecules form stacks along the *b*-axis, with adjacent molecules along the stack related to each other by translation. The plane of the aromatic ring system is tilted significantly with respect to the stacking axis (the angle between the normal to the aromatic plane and the *b*-axis is ca. 50.1°), and the perpendicular distance between the aromatic planes of adjacent molecules is ca. 3.80 Å (note that the plane of the aromatic ring is parallel to the *a*-axis). Adjacent stacks along the *c*-axis are related by the 2_1_-screw (parallel to the *b*-axis), and the planes of the aromatic rings of molecules in adjacent stacks form an equal but opposite orientation with respect to the *b*-axis, giving rise to the zigzag arrangement of molecular planes when viewed in projection along the *a*-axis (see Figure 3a). Adjacent stacks along the *a*-axis are related by translation (see Figure 3b) and therefore have identical molecular orientations. We note that (as seen from the view along the stacking axis in Figure 3b) the relative positions and orientations of molecules in adjacent stacks give rise to an efficient packing of the *tert*-butyl groups of adjacent molecules.

### Scanning Tunneling Microscopy

**Figure 4:**
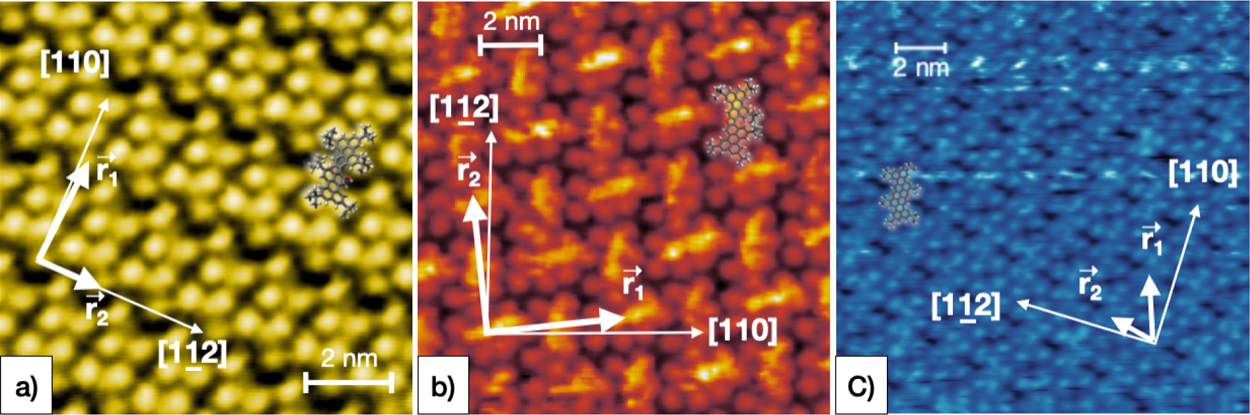
High-resolution constant current STM micrographs for ordered monolayers of **BPOL** (a), **BPF** (b), and **BPPP** (c) deposited on Cu(111). A geometric model of the compounds is superimposed to the topographic images as help to identify the single-molecule boundaries (identified by the peripheral six *tert*-butyl groups) and orientation. The images were recorded at room temperature using [80 pA; +220 mV], [90 pA; +260 mV], and [80 pA; −450 mV] for the tunneling junctions, respectively. The principal substrate directions **[110]** and **[112]** as well as the molecular unit vectors *r⃗*_1_ and *r⃗*_2_ are indicated as white arrows.

In Figure 4, high-resolution micrographs show the molecular features, as revealed by STM, on the **BPOL** (a), **BPF** (b), and **BPPP** (c) molecular networks assembled on the Cu(111) surface, with the corresponding molecular unit cell vectors [*r⃗*_1_, *r⃗*_2_, ϕ] reported in Table 1. From comparison of the STM images of the different molecules, the *tert*-butyl groups can be easily identified as protruding bright spots for all three molecules. In fact, the six *tert*-butyl groups allow us to discriminate easily the molecular boundaries and to determine quantitatively the molecular superlattices. Thus, we can confidently interpret the main intra-molecular contrast in terms of topographic effects.

**Table 1:** Molecular Superlattices Characterized by the Lengths |*r⃗*_1_| and |*r⃗*_2_| of the Unit Cell Vectors and the Angle ϕ between Them

	**BPOL**	**BPF**	**BPPP**
|*r⃗*_1_| [Å]	25.4 ± 0.2	27.3 ± 0.2	22.1 ± 0.2
|*r⃗*_2_| [Å]	15.2 ± 0.2	28.0 ± 0.2	16.2 ± 0.2
ϕ [deg]	89 ± 2	86 ± 2	58 ± 2

In Figure 4a, the relative intensity associated with the *tert*-butyl groups is not compatible with the strongly non-planar structure of the **BPOL** isolated molecule in the gas phase with the two perylene arms tilted by 63° with respect to each other.^24^ By looking carefully at the *tert*-butyl protrusions (six per molecule), we see a rather small difference in the height Δ*z*_MAX_ = 25–30 pm (see height distribution analysis in Supporting Information, Figure S11), also suggesting that perylenes should arrange in a flatter configuration than in the isolated gas-phase molecule. This refined analysis also matches the assumption of a more planar configuration described in the ARPES simulations, discussed below.

Regarding the adsorption geometry, **BPOL** is found to arrange in an approximately rectangular superstructure, while the **BPF** compound shows an almost square unit cell with a herringbone configuration. A rhomboidal brick-walled phase geometry describes the self-assembly for **BPPP**. We note that the **BPPP** arrangement bears some resemblance to the projection of the crystal structure of **BPPP** along the *b*-axis (Figure 3b), particularly regarding the presence of rows of molecules with their long axes essentially parallel to each other. However, the manner in which adjacent rows are displaced relative to each other is different, representing an alternative arrangement for packing the *tert*-butyl groups of adjacent molecules in the lateral direction compared to that observed (along the *a*-axis) in the crystal structure.

**Figure 5:**
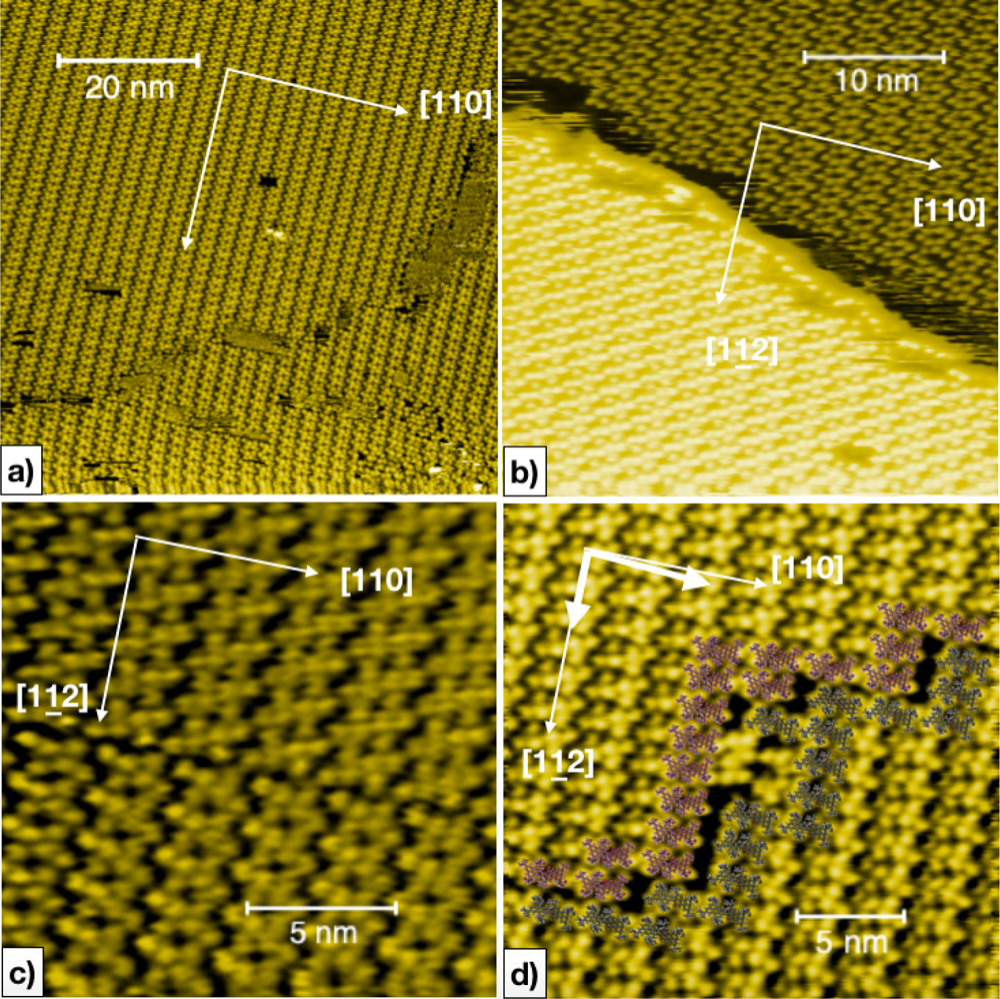
**BPOL** assemblies on Cu(111). (a) Long-range ordering with some defects at the border of chiral domains. (b) Step separating chiral domains on adjacent substrate terraces. (c,d) Domain walls between two distinct chiral assemblies. In (d), a cartoonic picture of the wall molecules (red- and green-colored for the two homochiral arrangements) is superimposed as an overlayer to guide the eye.

**Figure 6:**
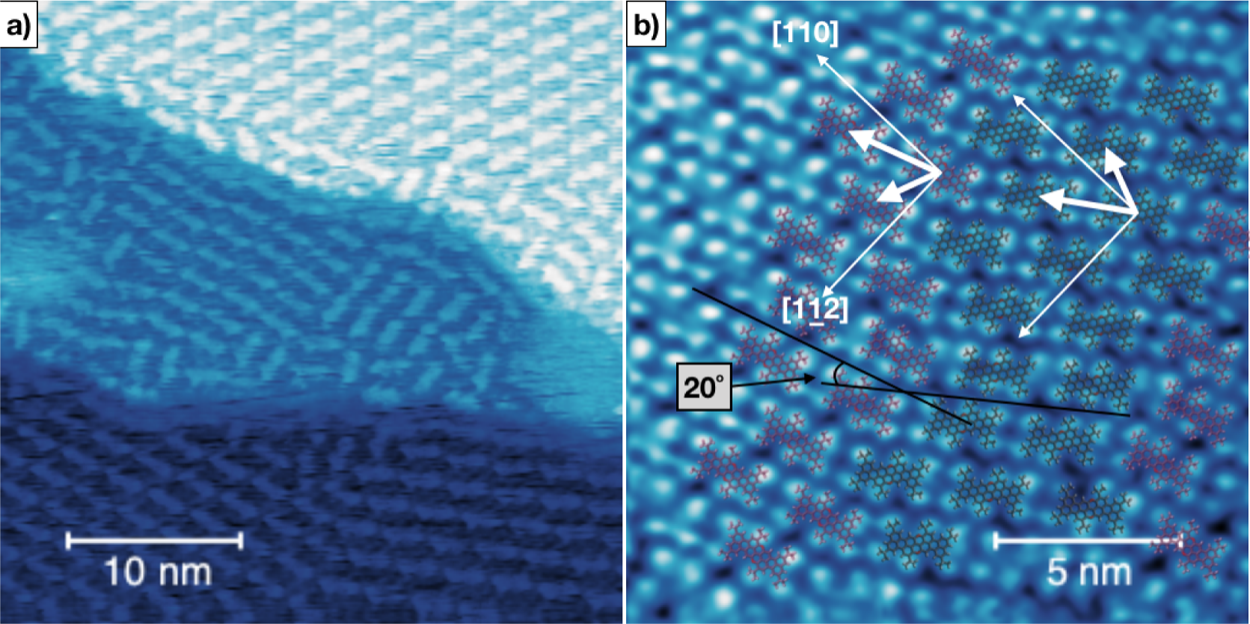
Molecular domains for **BPPP**/Cu(111). (a) Molecular ordering in the vicinity of the substrate’s steps is compromised, while extended molecular networks are formed a few nm away from the terrace edges. (b) Evidence of coexisting domains with opposite surface chirality. The superimposed molecular cartoonic pictures (red and green for the two surface enantiomers) help in the pattern visualization. From the combined analysis of the measured STM micrographs and LEED patterns, an angle of 20° between the two chiral partners is confirmed.

**Figure 7:**
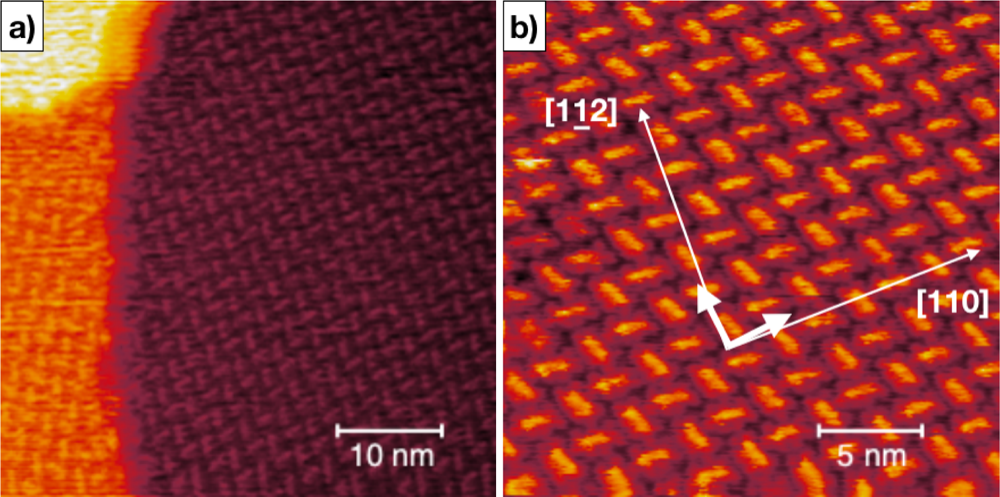
**BPF** arrangement on Cu(111). Low-resolution (a) and high-resolution (b) STM micrographs show the herringbone configuration for the square-like molecular pattern with no evidence of chiral domains.

In order to understand the chiral properties of the full-coverage monolayer films studied in this work, we note that we have used racemic mixtures, that is, equal amounts of left-handed and right-handed enantiomers, of the chiral molecules **BPOL** and **BPF**. The pro-chiral **BPPP** also shows self-organization into chiral domains upon adsorption. We have observed chiral domains, each one displaying three equivalent rotated domains according to the threefold symmetry of the Cu[111] substrate. The structures formed after the deposition of **BPOL** molecules, as shown in Figure 5, are characterized by two distinct geometric domains covering the substrate’s atomically flat terraces. The molecular domains extend for tens of nm (Figure 5a) and show no tendency of intermixing molecules with opposite chirality. Domains with opposite chirality are typically observed on the two sides of a substrate step, as in Figure 5b. Domain boundaries between opposite chiralities can however also be observed on terraces, as shown in Figure 5c,d. Besides the general tendency of molecules to decorate the substrate’s terrace edges by aligning their long axis parallel to the step, we have also observed the formation of chiral domains for **BPPP** (Figure 6a). The main difference with respect to **BPOL** is that this molecule does not have an intrinsic chirality, but shows a chiral assembly upon adsorption. We might argue that considering the deposition process at the molecular size, the energetic gain given by the close packing of **BPPP** molecules adsorbed with the same orientation can drive a similar adsorption in the nearby areas and/or induce a flipping for those adsorbed with opposite orientation (Figure 6b). In the case of **BPF** (Figure 7a,b), the intrinsic chirality does not clearly display different chiral domains by STM, and this is probably due to the square lattice adsorption geometry. Moreover crystallographic data reported for **BPF** and **BPOL** show a large decrease of the dihedral angle from 63° for **BPOL** to 17° for **BPF**, where the dihedral angle between the aryl moieties is drastically reduced by planarity of the furanyl framework,^24^ thus making **BPF** molecules adsorb flat on the surface with a further suppression of chirality-related effects.

### Low-Energy Electron Diffraction

**Figure 8:**
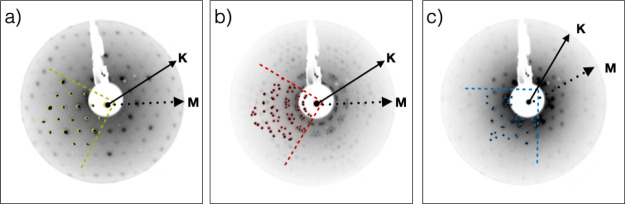
LEED diffractograms for 1 ML of **BPOL** (a), **BPF** (b), and **BPPP** (c) deposited on Cu(111) taken at electron energies of 24.0, 26.5, and 23.9 eV, respectively. Overlaid on the three images, we show a portion of the simulated pattern delimited by dashed lines as well as the substrate’s high-symmetry directions (**K** and **M** corresponding to **[110]** and **[112]**, respectively).

Figure 8 displays the LEED diffractograms of the three molecular species, taken in the energy range of 23–27 eV, after depositing 1 ML on Cu(111). The pattern visible in this energy range corresponds to the large molecular unit vectors as measured by STM and reported in Table 1, while the substrate Cu(111) pattern (unit vector *a⃗* = 2.58 Å, whose spots have larger separation in the reciprocal space) can be appreciated for *E* > 50 eV (See Figure S10 in Supporting Information). The LEED pattern simulation with the given parameters allows comparison with the experimental data. Considering the threefold *p*3*m*1 symmetry for the Cu(111) substrate, we have considered the coexistence of six equivalent domains for each of the three molecular lattices (rectangular for **BPOL**, square for **BPF**, and rhomboidal for **BPPP**). In Figure 8, the superimposed simulated spot distribution (delimited by the dashed lines) is in good agreement with the experimental results and designates the incommensurate molecular lattices. Using the parameters obtained by STM (Table 1) as input for the LEED superstructure evaluation, we obtain the matrices

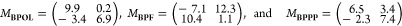
(matrix elements reported with an error of ±0.1). The corresponding areas of the molecular superlattice are found to be 386 ± 8 Å^2^, 764 ± 10 Å^2^, and 310 ± 7 Å^2^ for **BPOL**, **BPF**, and **BPPP**, respectively.

### Photoemission Results

**Figure 9:**
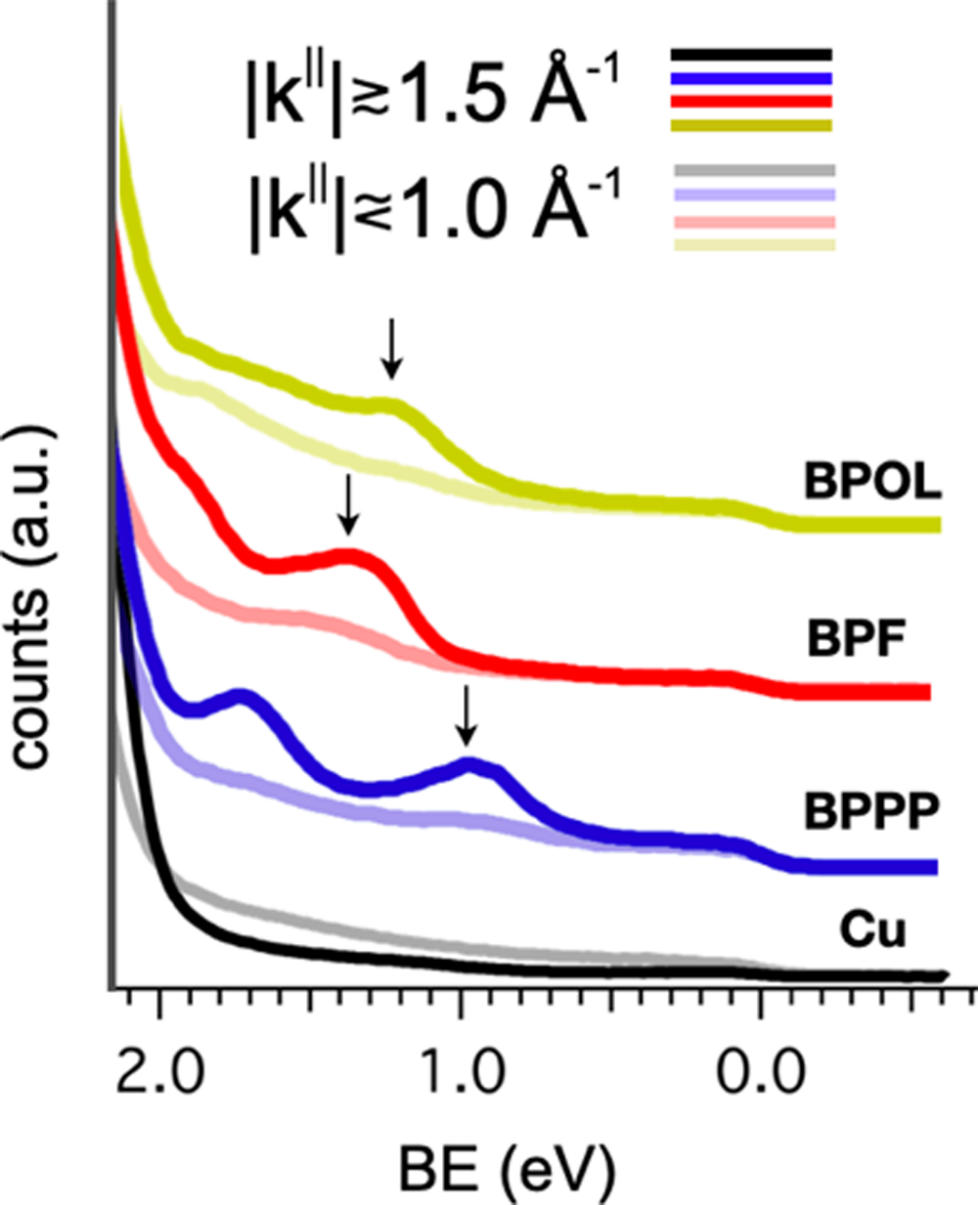
Integrated UPS spectra taken at |**k**^∥^| values of 0.8 Å^–1^ (half transparent traces) and 1.5 Å^–1^ (fully colored traces). Molecular-related features (HOMOs are indicated by arrows) are present in the binding energy range of 0.9–2.0 eV (labeled traces **BPOL**, **BPF**, and **BPPP**), while the spectrum taken on the clean Cu substrates is flat.

In Figure 9, we show that for *k*^∥^ values of approximately 0.8 Å^–1^, following the curves from the Fermi level to higher binding energies, no structures are present before the substrate states, while the story is different at 1.5 Å^–1^ (half transparent vs fully colored lines). The HOMO positions are found at 1.24, 1.40, and 1.00 eV for **BPOL**, **BPF**, and **BPPP** (yellow, red, and blue curves, respectively). In addition, for the **BPPP** case, a clear peak is found at a binding energy of 1.80 eV, which is tentatively attributed to the HOMO – 1 level. No evidence of LUMO-filled states in the vicinity of the Fermi level together with the large HOMO–LUMO gaps for the same compounds, as has been reported by Miletić et al.,^23^ indicates a limited charge transfer from the substrate with a possible band alignment of the substrate Fermi level in the middle of the surface HOMO–LUMO gap.

A number of experimental and theoretical works on different molecular structures have been published by following the orbital tomography methodology.^15,31,55−60^ In our case studies, we partially meet the requirements for the PW final-state assumption since we show experimental data on quasi-planar and planar self-assembled large π-conjugated molecules. The deviation from strictly planar molecules may be the origin of the discrepancy between the experimental and simulated results.

Photoemission simulations have been carried out for the isolated molecules within the PW approximation, with the initial state being supplied by DFT calculations (i.e., the well-known photoemission tomography method^14,61^). The geometric structures of gas-phase **BPPP** and **BPF** are comparatively flat, so we neglected the inevitable change of geometry upon adsorption on the substrate. Gas-phase **BPOL** is, however, non-planar, and significant changes upon adsorption on the Cu(111) surface are expected. The STM images in Figure 4 also appear to indicate this, and we think that the flattened (even if not completely) molecule (also shown in Figure 5) is in better agreement with the STM measurements. As can be seen from the EDC in Figure 9, two peaks are discernible for **BPPP** (which we assign to the HOMO and HOMO – 1 states), while there is only one peak for **BPF** and **BPOL** (which we assign to their corresponding HOMO levels). By taking into account the geometrical arrangement deduced by the combined STM and LEED analysis, we have proceeded in the orbital map reconstruction as follows.

**Figure 10:**
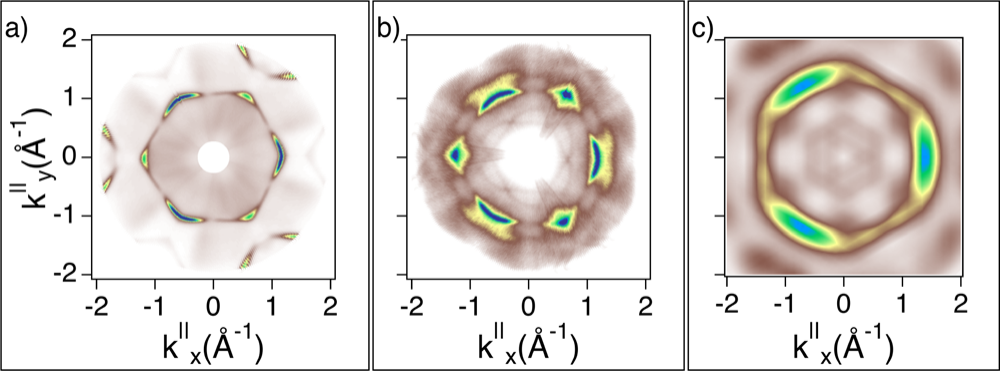
Constant energy cut at BE = 1.24 eV on the clean Cu(111) (a) and for **BPOL**/Cu(111) (b). The corresponding HOMO simulation is shown in (c). All plots have been oriented to display *k*_*x*_^∥^ and *k*_*y*_^∥^ aligned along the substrate’s high-symmetry directions (**M** and **K** corresponding to **[112]** and **[110]**, respectively).

The results summarized in Figure 10 describe the case of the **BPOL** molecule for which we must consider two enantiomers with their corresponding lattice domain orientations; since the long molecular axis for both enantiomers is parallel to the [110] direction, the corresponding overall HOMO map is given by summing the single oriented orbital maps with the rotated ±120° accounting for the threefold substrate’s symmetry (symmetrization). The constant energy cuts are compared to the clean substrate (shown in Figure 10a). The match between the simulated and experimental maps (Figure 10b,c) is acceptable if we consider that in this case, the non-planar adsorption invalidates the assumptions of orbital tomography. In the experimental map in Figure 10b, we observe interface states, also visible on the Fermi surface (*E*_b_ = 0 eV) from features of substrate photoelectrons diffracted by the molecular lattice. These features have already been described, as a result of final-state effects, for other π-conjugated molecules assembled in ordered networks^62,63^ (see Figure S2 in Supporting Information).

In the **BPF** case, LEED and STM results confirm the hypothesis of a square-like pattern with herringbone layout and and a slight (∼5°) misorientation from the [110] direction. If we consider that in both chiral herringbone domain lattices the two molecules are adsorbed with 60° angular displacements, we can construct the overall photoemission map by summing up the four single-molecule orbital maps (0–60°), two +5° and two −5°, rotated with respect to the [110] direction and then proceed with the threefold symmetrization (details in Figure S4). Also in this case, the small discrepancy between the experimental and simulated maps can be ascribed to the non-flatness of the molecules even if this aspect is not as strong as for **BPOL**, with a better agreement between experiment and simulation, as shown in Figure 11.

**Figure 11:**
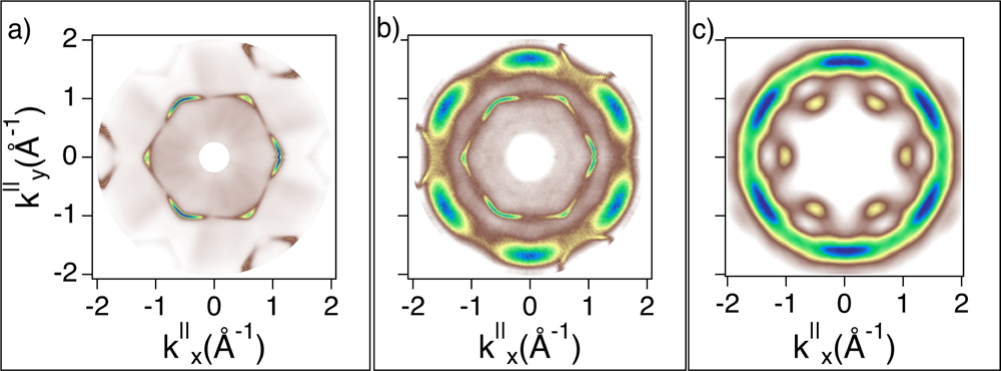
Constant energy cut at BE = 1.4 eV on the clean Cu(111) (a) and for **BPF**/Cu(111) (b). The corresponding HOMO simulation is shown in (c). All plots have been oriented to display *k*_*x*_^∥^ and *k*_*y*_^∥^ aligned along the substrate’s high-symmetry directions (**M** and **K** corresponding to **[112]** and **[110]**, respectively).

**Figure 12:**
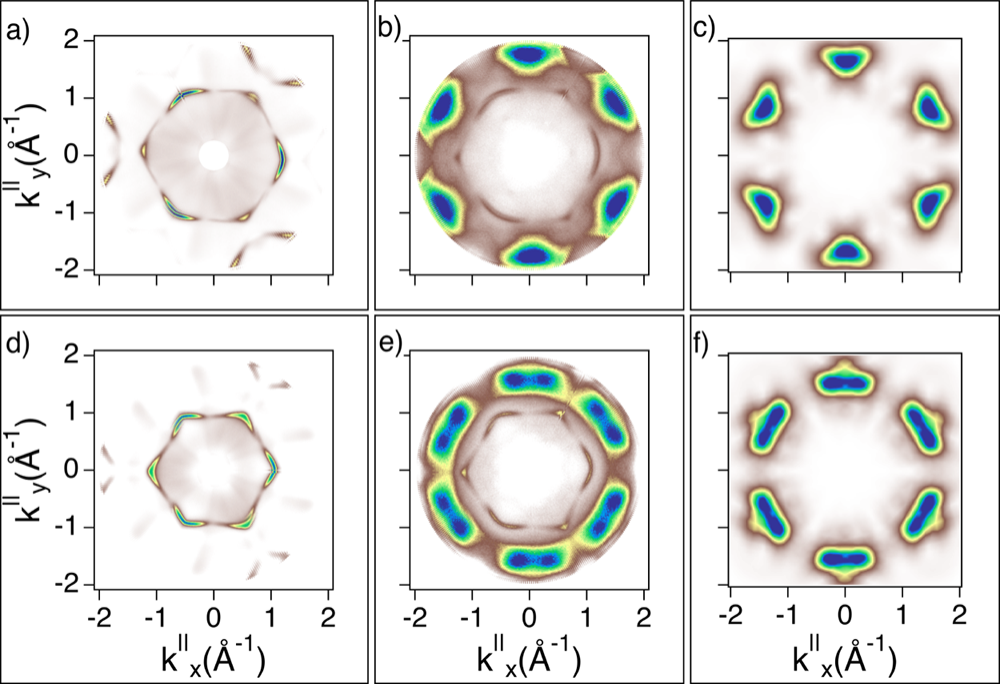
Constant energy cut at BE = 1.0 [1.8] eV on the clean Cu(111) (a,d) and for **BPPP**/Cu(111) (b,e). The corresponding HOMO [HOMO – 1] simulation is shown in (c,f). All plots have been oriented to display *k*_*x*_^∥^ and *k*_*y*_^∥^ aligned along the substrate’s high-symmetry directions (**M** and **K** corresponding to **[112]** and **[110]**, respectively).

The case of **BPPP** is peculiar and worth discussing in some detail. The two pro-chiral forms adsorb on the surface forming two distinct domains (see Figure 6) with the same superlattice parameters (**BPPP** column of Table 1) but with different orientations with respect to the substrate high-symmetry directions. In particular, there is a 20° angle between the longer axis of one chiral lattice and that of the mirror one. With *L* and *R* enantiomers being the two pro-chiral partners, they must be oriented with their long molecular axis rotated clockwise by 20° and 40°, respectively. Inversely, if one considers anti-clockwise rotations, the comparison of the experimental and simulated patterns gives a full correspondence, as shown in Figure 12, only if *L* is rotated by 40° and *R* by 20°. A schematic for *L* and *R* orientations with details on the map reconstruction is shown in Supporting Information (Figures S5 and S6). Referring to this analysis, we conclude that the *L*(*R*) molecules adsorb with a sixfold symmetry with a clockwise rotation of 20°(40°) from the [110] substrate direction.

As can be seen from Figures 10, 11, and 12, the gas-phase simulations match the main features of the measured momentum maps. It is worth noting that there is a slight geometrical deviation of the simulated maps (sixfold symmetric) with respect to the measured ones (threefold symmetric). For the simulations, the substrate is taken into consideration only for the surface symmetry taken as a reference for the molecular orientation. It is therefore reasonable to obtain a sixfold symmetry as the molecule is twofold symmetric, at least approximately. To some extent, the sixfold symmetry is also visible in the measured maps, where the contribution of the threefold symmetric underlying surface states cannot be disentangled from those of the molecular orbitals. This means that we must take into consideration that the emission of the overlayer is modified due to hybridization with the substrate. In addition, the moderate agreement between the calculated and experimental maps together with the EDC of Figure 9 indicates that the LUMO remains empty upon adsorption.

## Conclusions

In summary, we have demonstrated that, by means of surface science methodologies combining structural and electronic state information, it is possible to characterize the electronic structure of large molecules that are able to assemble in large ordered networks. In particular, we have investigated O-doped PAHs (**BPOL**, **BPF**, and **BPPP**) in which fine tuning of both the π-extension of the carbon scaffold and the oxygen linkages is responsible for slight modifications of the HOMO and LUMO state positions. STM investigation of this class of molecules has revealed the presence of chiral domains, and the patches composed of single enantiomers do not intermix, indicating the mutual recognition of molecules during the self-assembling process.

The position in the energy and parallel momentum space of the HOMOs has been revealed with no direct information on the HOMO–LUMO gaps since no LUMO filling has been observed after the absorption on the Cu(111) substrate. Nevertheless, considerations on the gap measured for the same compounds under other conditions suggest the possible molecular band alignment with the substrate Fermi level in the middle of the molecular gap. Moreover, the tunability that can be achieved by synthetic modification of the compound geometry allows a possible benchmark for the reconstruction methods of molecular orbitals from photoemission data.

Eventually, by means of the PW approximation or more sophisticated methodologies, it would be possible to use the experimental electronic fingerprints measured by orbital tomography to reconstruct the density of states of the molecular network and relate it with the actual adsorption phase including the possible hybridization with the substrate which in turn can modify its electronic structure in response to the overlayer.

## Supporting Information Available

Additional details on orbital mapping, LEED, and STM (PDF)

## Notes

The crystal structure of **BPPP** has been deposited as a cif file in the Cambridge Structural Database (CSD) deposition number 2078994.

## Footnotes

a The software utility LEEDpat, Version 4.2, (C) K.E. Hermann (FHI Berlin) and M.A. Van Hove (Baptist University Hong Kong), was used in the evaluation of the data; see also http://www.fhi-berlin.mpg.de/KHsoftware/LEEDpat/index.html.
