# Supplementary material for: Orbital Mapping of Semiconducting Perylenes on Cu(111)
Source: J Phys Chem C Nanomater Interfaces. 2021 Oct 28;125(44):24477–86. doi: 10.1021/acs.jpcc.1c05575 (PMC8592032; doi:10.1021/acs.jpcc.1c05575)
Supplement: Supplementary file 1 — jp1c05575_si_001.pdf [file jp1c05575_si_001.pdf]

Orbital Mapping of Semiconducting Perylenes on Cu(111)

Giovanni Di Santo,[*,†] Tanja Miletić,[‡] Mathias Schwendt,[¶] Yating Zhou,[‡] Benson M. Kariuki,[‡] Kenneth D. M. Harris,[‡] Luca Floreano,[§] Andrea Goldoni,[†] Peter Puschnig,[¶] Luca Petaccia,[†] and Davide Bonifazi[‖,‡]

†*Elettra Sincrotrone Trieste, Strada Statale 14 km 163.5, 34149 Trieste, Italy*

‡*School of Chemistry, Cardiff University, Park Place, CF10 3AT, Cardiff (UK)*

¶*Institute of Physics, University of Graz, NAWI Graz, 8010 Graz, Austria*

§*CNR-IOM Lab. TASC in Area Science Park, s.s. 14, Km 163.5 - 34149 Trieste, Italy*

‖*Institute of Organic Chemistry, University of Vienna, Währinger Str. 38, 1090 Vienna, Austria*

E-mail: giovanni.disanto@elettra.eu

Supplementary Information

Orbital mapping details

In Figure S1 we show the results of the ARPES experiments in form of constant binding energy slices of the photoemission data cube $I(E_b, k_x, k_y)$ by comparing the clean Cu(111) substrate (a,e,i,m) with the molecular films (b,f,j,n) for **BPOL**, (c,g,k,o) for **BPF** and (d,h,l,p) for **BPPP**. At binding energies of 1.0, 1.4 and 1.8 eV, there are distinct features ascribed

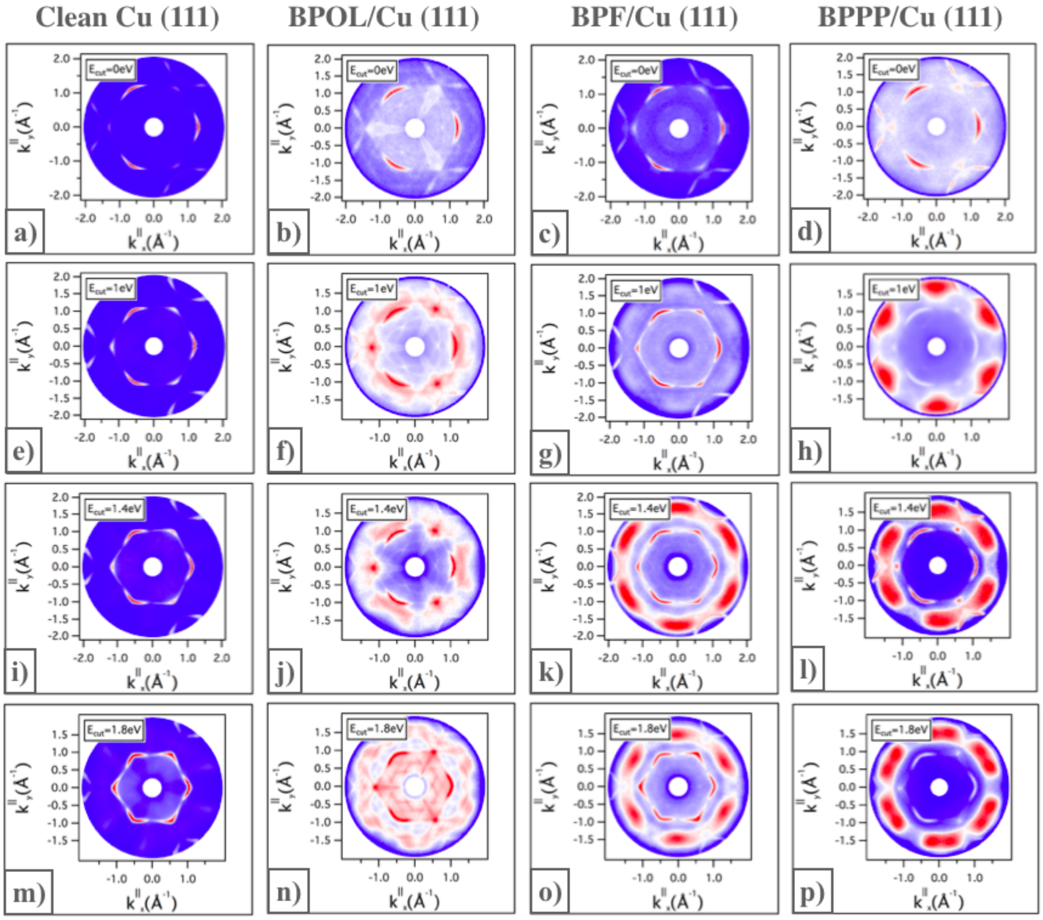

Figure S1: Results of ARPES experiments, performed with an excitation photon energy of 31 eV, in form of parallel momentum maps at different binding energies. For each energy cut (rows) it is shown the corresponding symmetrized map (columns) for the clean Cu(111) and the 1ML(mol)/Cu(111). By rows: Fermi surfaces (a,b,c,d) and binding energy slices (e,f,g,h) at 1.0 eV, (i,j,k,l) at 1.4 eV, (m,n,o,p) at 1.8 eV

to the presence of the highest occupied molecular states (HOMOs), with a weaker intensity

for **BPOL** with respect to **BPF** and **BPPP**. Distinct intensity for molecular states (lobes) are present at $k^{||}$ values larger than 1 Å^{-1}. In the case of **BPF** and **BPPP**, the momentum distribution of photoemission intensity is most clearly visible and localized at $|k^{||}| \simeq 1.5$Å^{-1}. The geometric position of the lobes in the k_x, k_y plane with respect to the substrate high symmetry directions is consistent with the incommensurate lattice geometries found in the LEED experiments. At binding energies higher than 2.0 eV, where the substrate states are predominant, deeper molecular states are overlapping with the substrate's resulting in a mild modifications (blurring) of the momentum space metal band structure. For **BPOL** we have

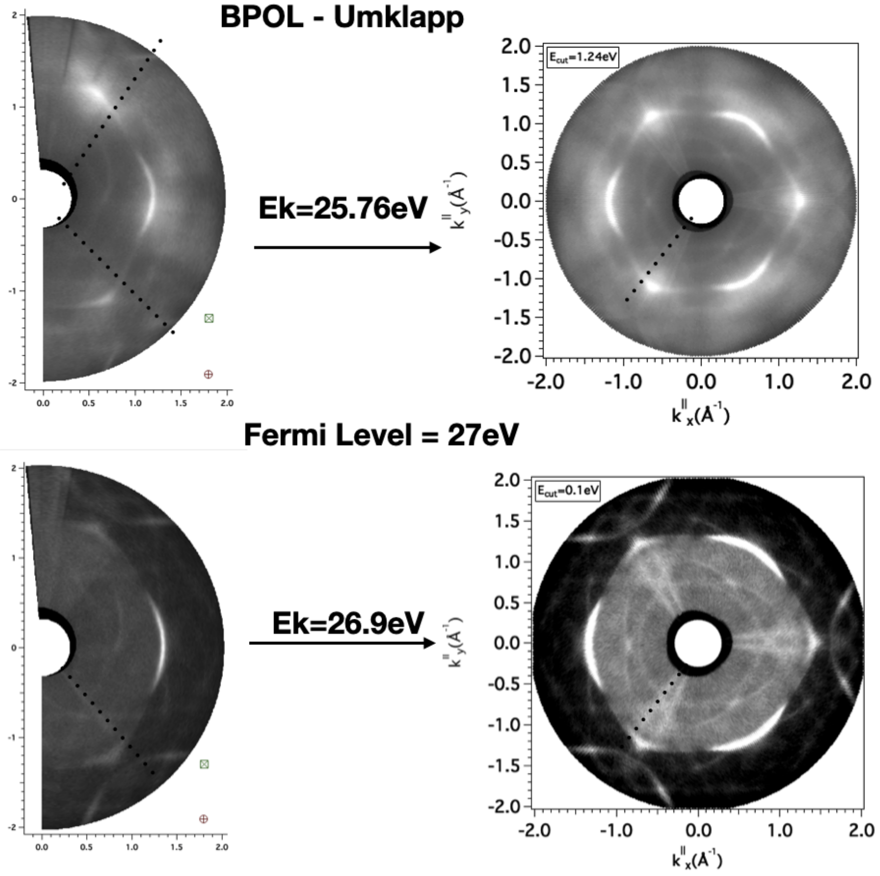

Figure S2: Unsymmetrized (left) and symmetrized (right) constant energy photoemission maps of **BPOL**/Cu(111) taken at KE=25.76 eV assigned to HOMO states (top) and at KE=26.9 eV (bottom). The non radial pattern is attributed to the substrate states diffracted by the molecular lattice while the radial features (indicated by dotted lines) are artifacts due to intensity mismatch between subsequent azimuthal steps in the maps.

observed final states effects due to substrates features diffracted by the molecular network

(umklapp) shown in figure S2 for two constant energy cuts. The non radial lines are assigned to the diffraction from substrates features with the specific threefold geometry, while Radial features visible in both the unsymmetrized and symmetrized maps have to be considered as experimental artifacts due to intensities unbalance between subsequent azimuthal steps (1°) during the map acquisition.

In the following figures S3, S4, S5 and S6 we summarize the simulated map reconstruction details for the three compounds.

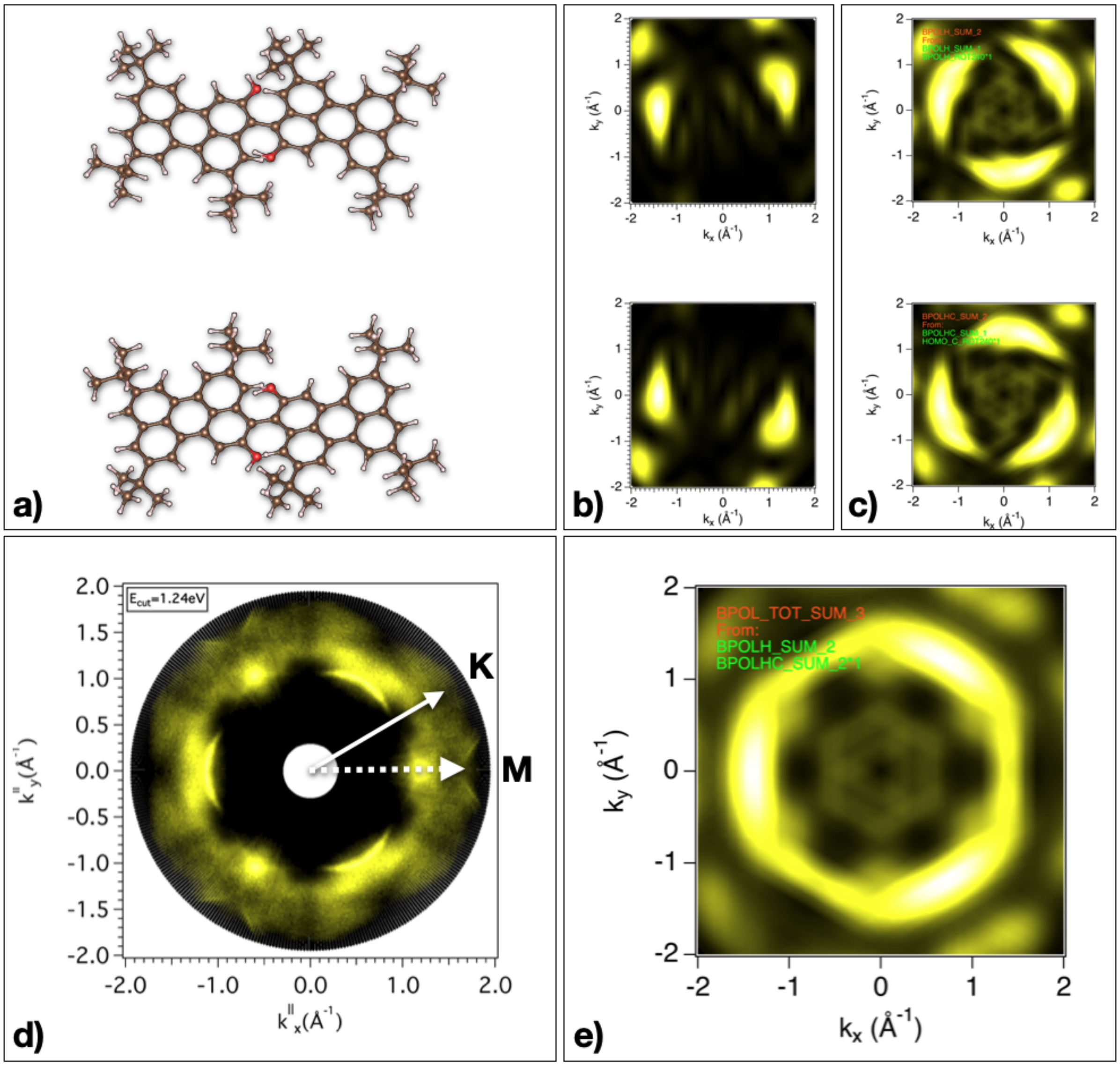

Figure S3: Overview on the calculated photoelectron momentum HOMO map construction for **BPOL**. a) Cartoonic pictures of the two chiral enantiomers and b) their corresponding HOMO maps. c) HOMO maps for the two chiralities after three-fold symmetrization. d) Experimental photoemission map ($E_{HOMO} = 1.24\ eV$). e) Simulated map obtained by summing the two symmetrized images in c).

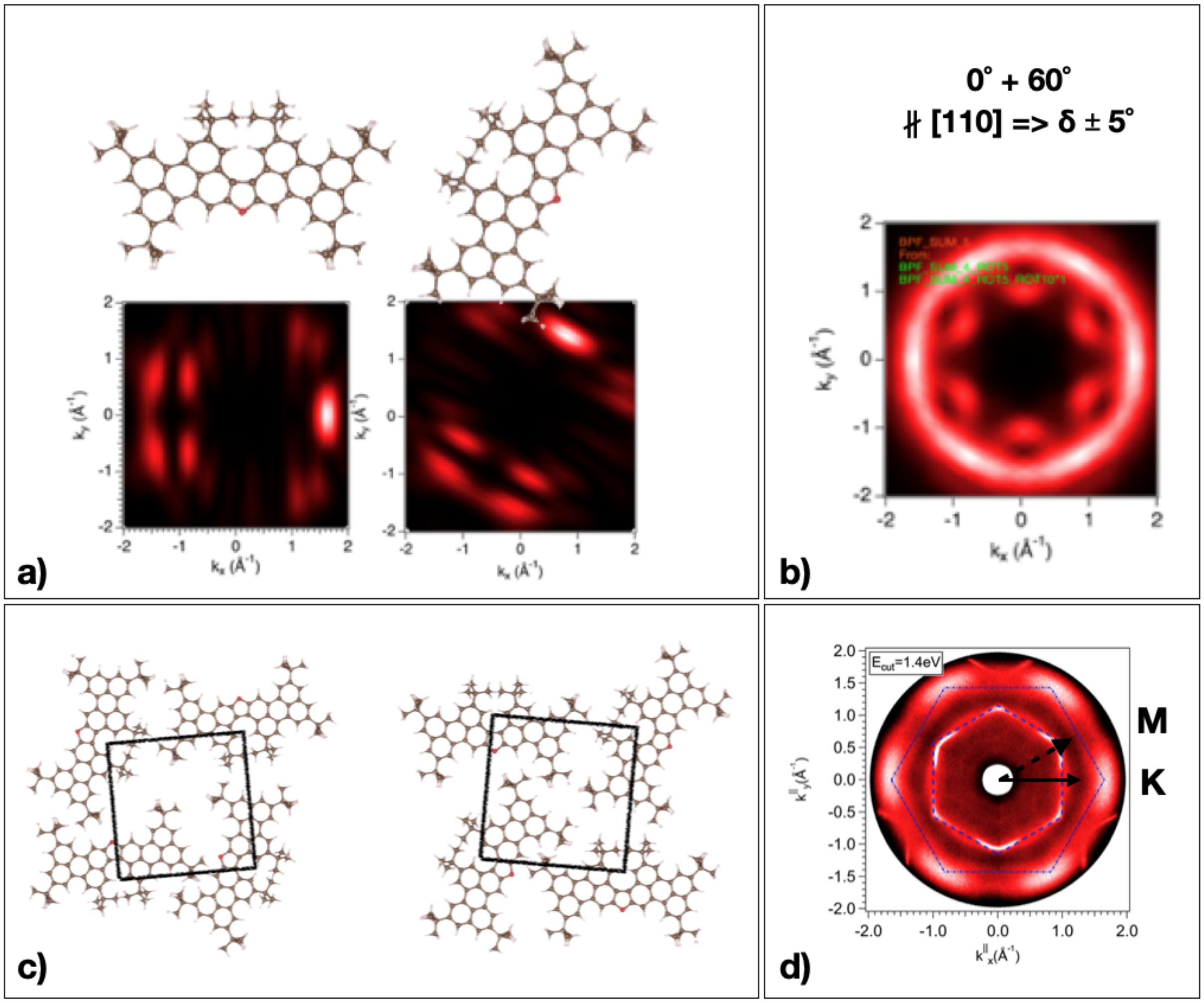

Figure S4: Overview on the calculated photoelectron momentum HOMO map construction for **BPF**. a) Cartoonic pictures of the **BPF** molecule and its corresponding HOMO map parallel (0°) and 60° rotated with respect to the [110] direction. b) Sum of the contributions, including threefold symmetry operations, for the two chiral lattices (schematically drawn in c)) which have ± 5° misalignment with respect to the [110] direction. d) Experimental photoemission map ($E_{HOMO} = 1.4\ eV$).

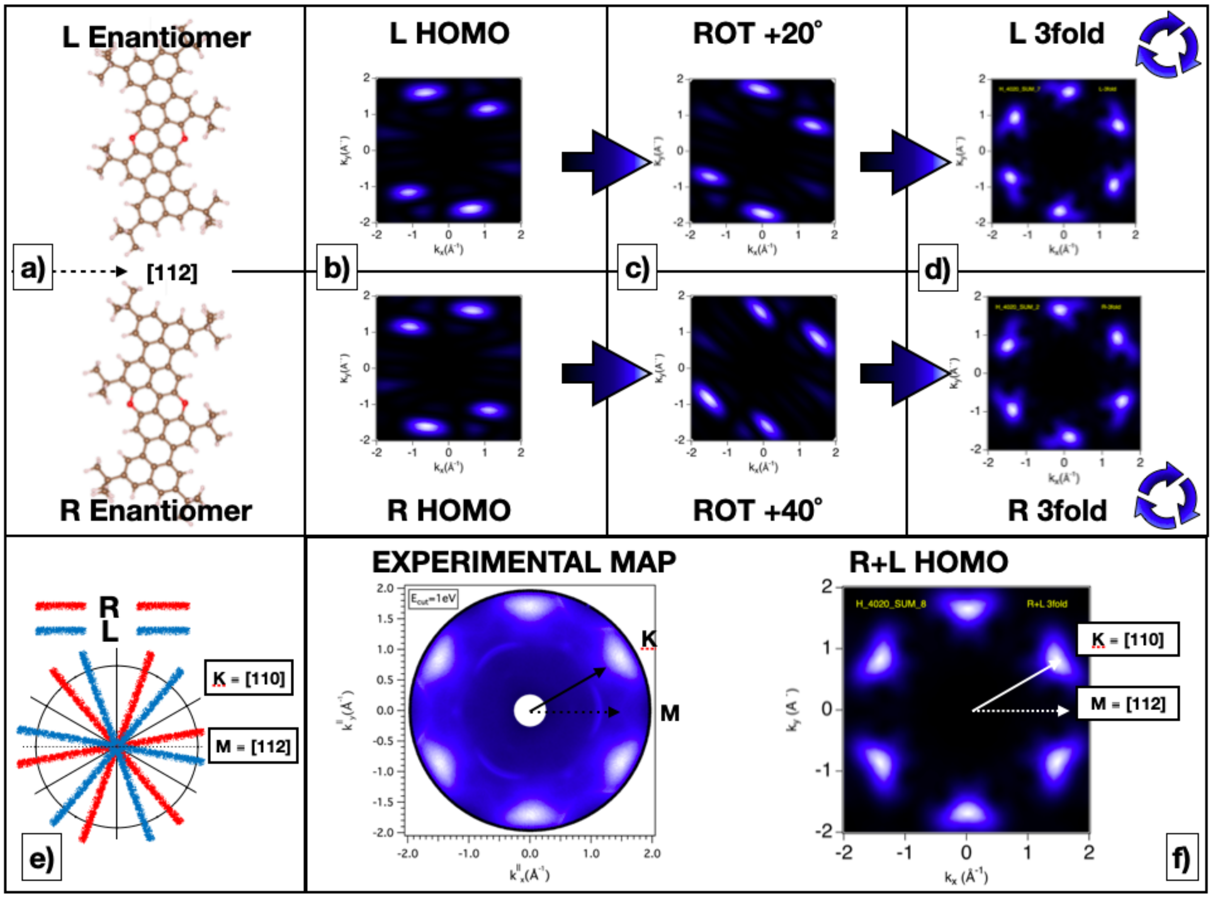

Figure S5: Overview on the calculated photoelectron momentum HOMO maps construction for **BPPP**. a) real space image of the two pro-chiral enantiomers and b) their corresponding HOMO maps. c) HOMO maps for the two chiralities and d) after three-fold symmetrization according to the orientation angles (for L +20° and for R +40°) inferred by combined STM and LEED analysis. In e) a schematics showing the discovered molecular orientations for L and R types with respect to the substrate. f) Comparison between the experimental photoemission map (E_{HOMO} = 1.0 eV) and the simulated one taking into account the overall chiral contribution.

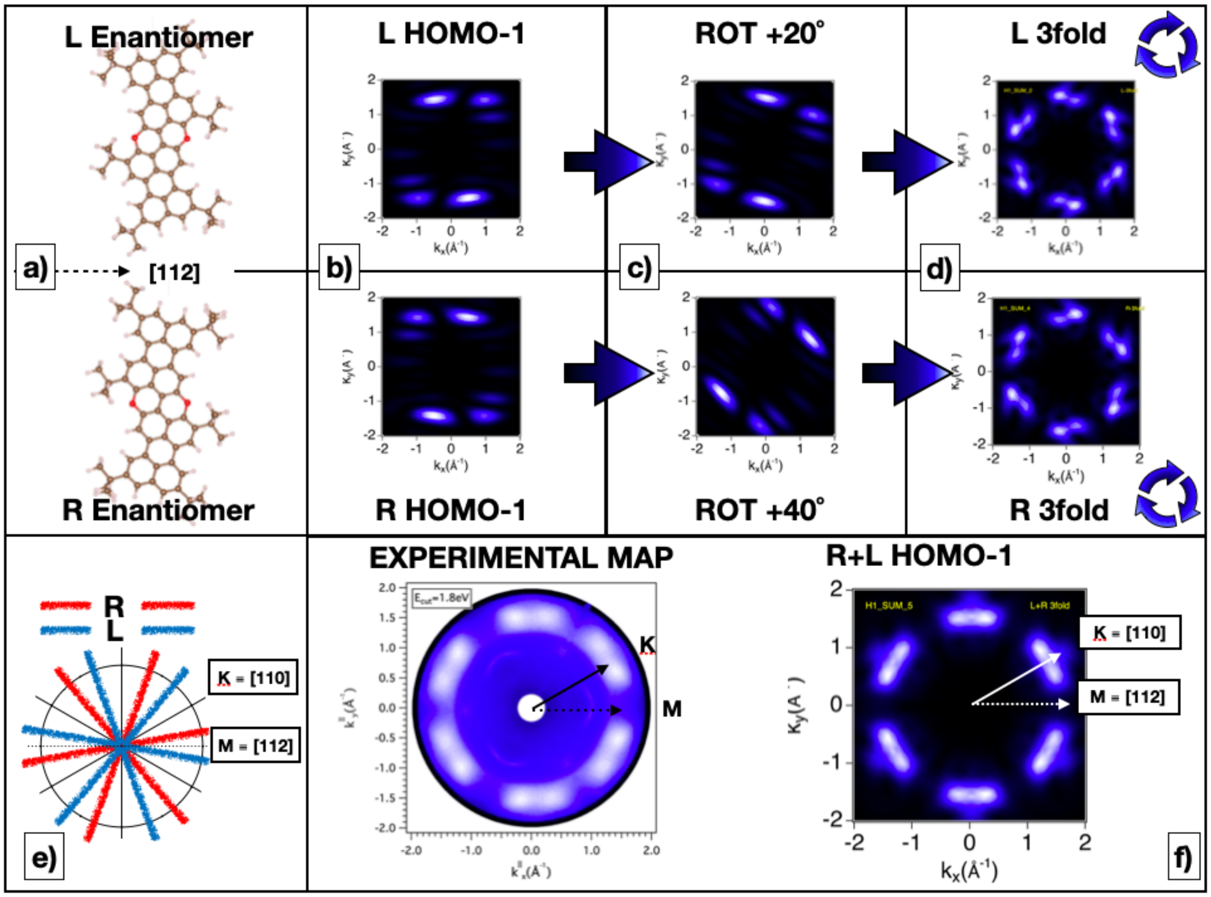

Figure S6: Overview on the calculated photoelectron momentum HOMO-1 maps construction for **BPPP**. a) real space image of the two pro-chiral enantiomers and b) their corresponding HOMO-1 maps. c) HOMO-1 maps for the two chiralities and d) after three-fold symmetrization according to the orientation angles (for L +20° and for R +40°) inferred by combined STM and LEED analysis. In e) a schematics showing the discovered molecular orientations for L and R types with respect to the substrate. f) Comparison between the experimental photoemission map ($E_{HOMO-1} = 1.8\ eV$) and the simulated one taking into account the overall chiral contribution.

LEED details

In this section we present additional figures S7, S8 and S9 for better explaining how we assign the matrix for the correspondence between the substrate and the molecular overlayer. In particular using the LEEDPath software we can simulate the overall LEED pattern, including all the equivalent domains, using the substrate symmetry and the input superlattice vectors measured by STM. Then, by rotating the superlattice mesh with respect to the substrate one, we find the pattern, and the corresponding matrix, which is best matching the experimental LEED images. In figure S10 we present a summary of the pictures acquired at different energies for visualizing the substrate ($E \geq 70\ eV$) and over-layer ($E \leq 30\ eV$) patterns.

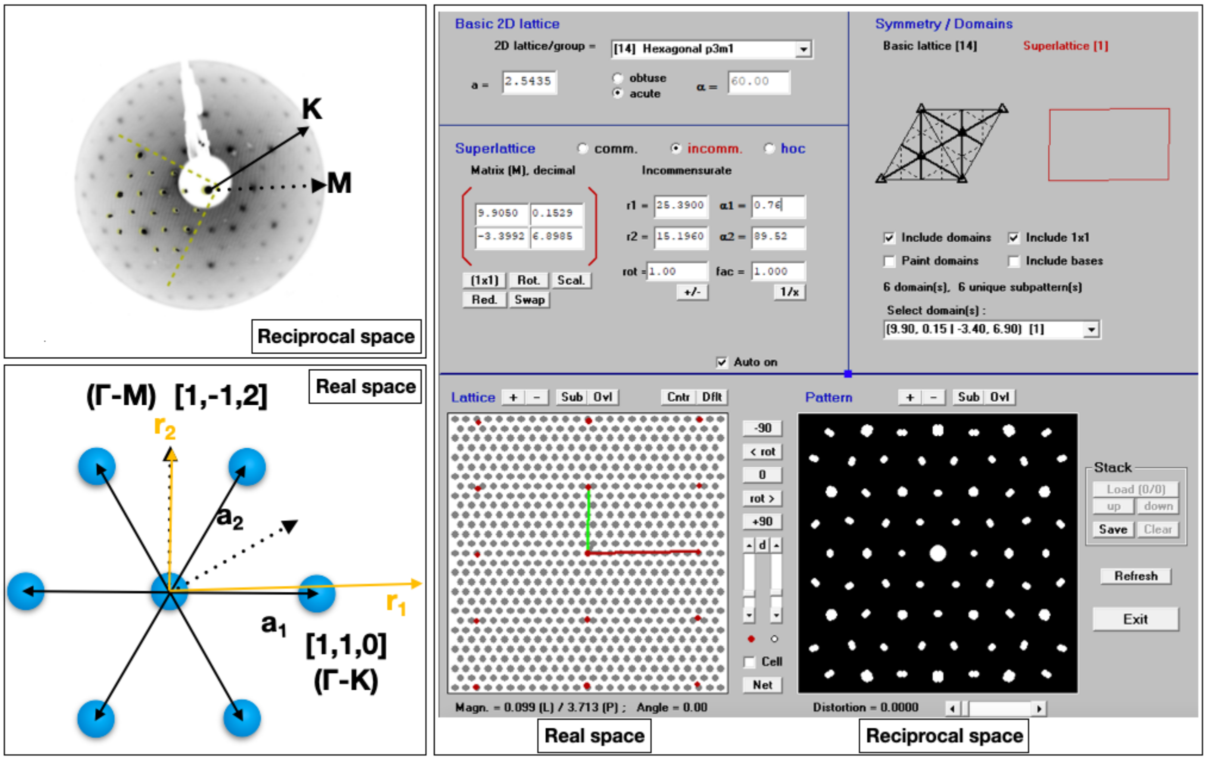

Figure S7: Summary of the LEED analysis results for **BPOL**. Left panels: Experimental LEED pattern acquired at 24.0 eV (top) and schematic picture for the orientation in real space of the substrate's and superlattice's vectors (bottom). Right panel: screenshot of the LEED analysis program (LEEDPath) with the simulated reciprocal space pattern obtained using the vector lenghts measured in STM and rotating the whole superlattice until the best matching with the experimental pattern is found.

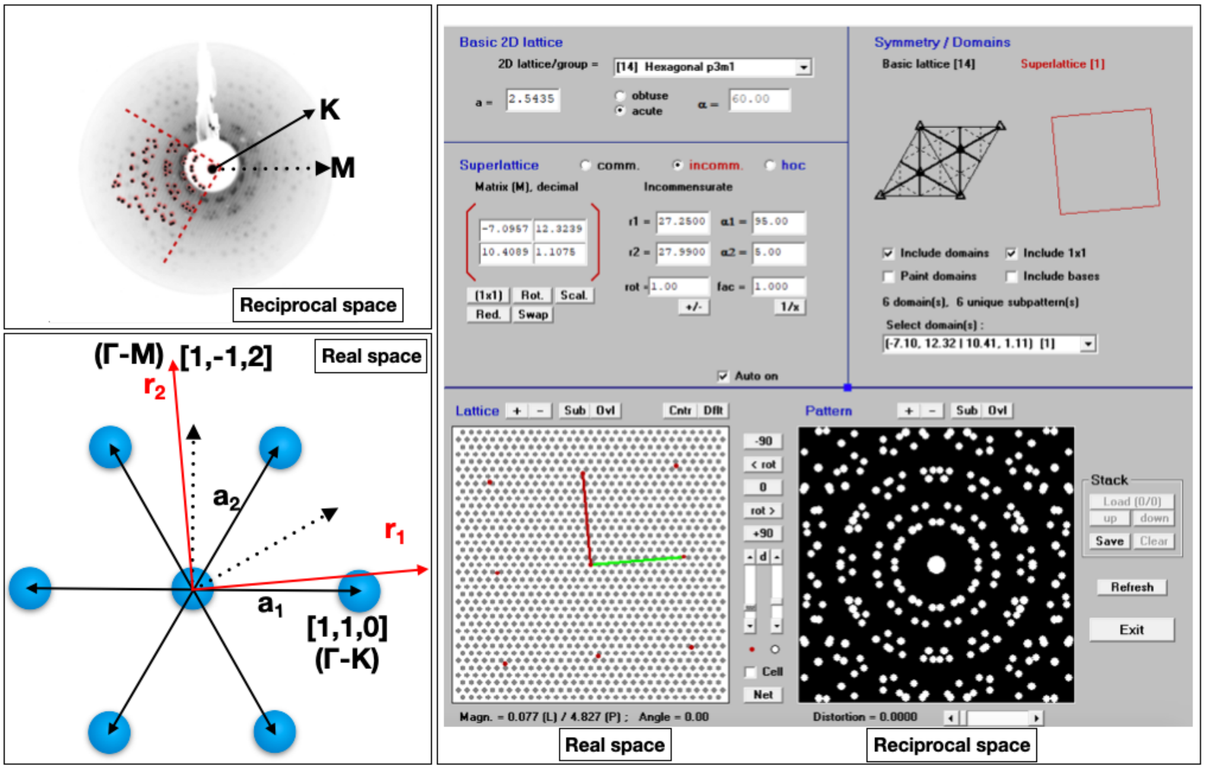

Figure S8: Summary of the LEED analysis results for **BPF**. Left panels: Experimental LEED pattern acquired at 26.5 eV (top) and schematic picture for the orientation in real space of the substrate's and superlattice's vectors (bottom). Right panel: screenshot of the LEED analysis program (LEEDPath) with the simulated reciprocal space pattern obtained using the vector lenghts measured in STM and rotating the whole superlattice until the best matching with the experimental pattern is found.

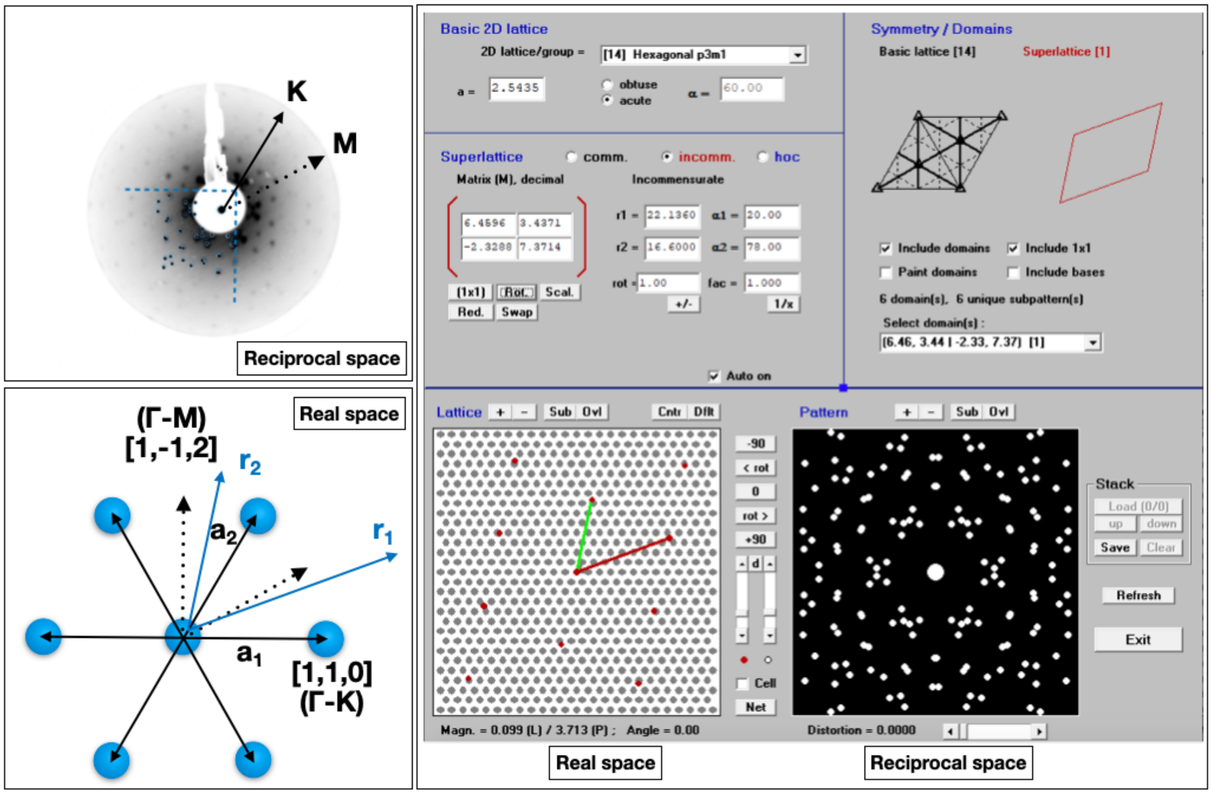

Figure S9: Summary of the LEED analysis results for **BPPP**. Left panels: Experimental LEED pattern acquired at 23.9 eV (top) and schematic picture for the orientation in real space of the substrate's and superlattice's vectors (bottom). Right panel: screenshot of the LEED analysis program (LEEDPath) with the simulated reciprocal space pattern obtained using the vector lenghts measured in STM and rotating the whole superlattice until the best matching with the experimental pattern is found.

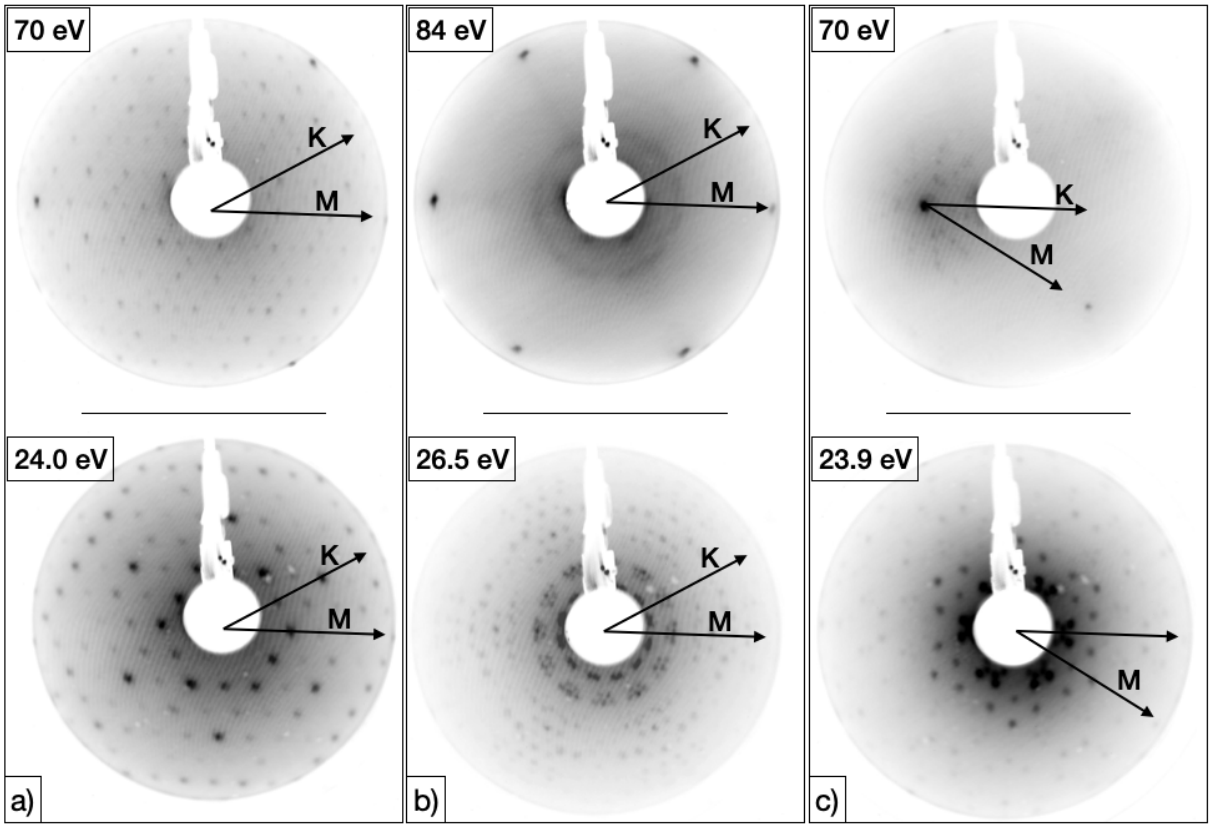

Figure S10: Summary of the LEED patterns acquired for **BPOL**(a), **BPF**(b) and **BPPP**(c). In each panels the top picture is acquired at $E \geq 70\ eV$ showing the substrate's spots and a faint over-layer pattern. The bottom pictures, acquired at $E \leq 30\ eV$ are the optimal for the over-layer.

STM details

In fig.S11 we show the main protrusions height distribution extracted from the STM micrograph acquired on **BPOL**/Cu(111). Each protrusion, marked using a watershed algorithm, is associated to a tert-butyl group (six per molecule) which act as markers for the molecule boundaries. The histogram show the height maxima in the 55-85 pm range, so with a maximum difference of 30 pm over $\sim$ 1 nm lateral size within the same molecule; we therfore obtain a tilt angle of $\leq$ 2° for the corresponding plane with respect to the surface. Even if this plane is only indirectly related to the perylen one we can reasonably assume that also the perylens are not far from a flat configuration.

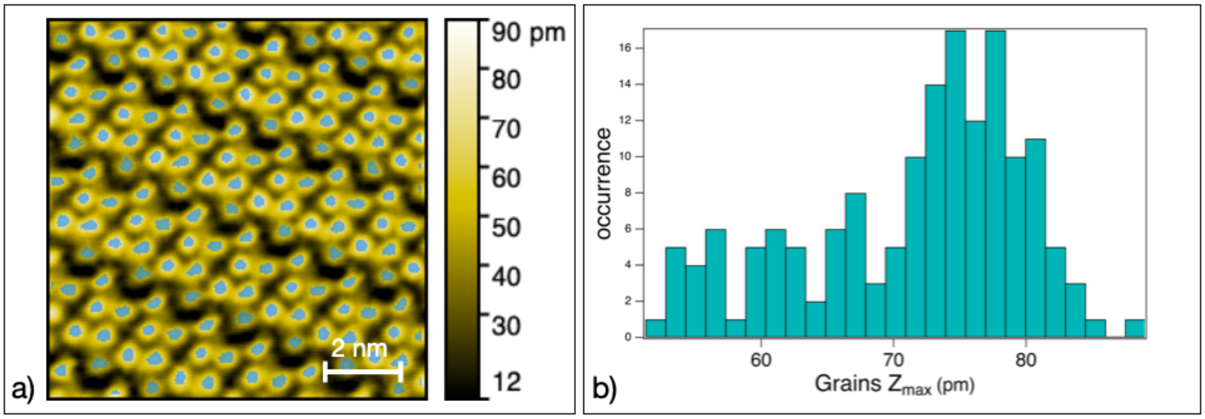

Figure S11: Height analysis from the STM image acquired for **BPOL**. Left panel: STM image with superimposed mask for particle marking obtained by using a watershed algorithm. Right panel: histogram of the height constructed by counting the occurrence of the local maxima, i.e. z_{MAX} of each marked grain. The height distribution indicate the largest mismatch to be within 30 pm.
